# Sexually Dimorphic Behavioral and Genetic Outcomes Associated With Administration of TA65 (A Telomerase Activator) Following Repetitive Traumatic Brain Injury: A Pilot Study

**DOI:** 10.3389/fneur.2020.00098

**Published:** 2020-02-18

**Authors:** Eric Eyolfson, Haris Malik, Richelle Mychasiuk

**Affiliations:** ^1^Department of Psychology, Alberta Children's Hospital Research Institute, Hotchkiss Brain Institute, The University of Calgary, Calgary, AB, Canada; ^2^Department of Neuroscience, Central Clinical School, Monash University, Melbourne, VIC, Australia

**Keywords:** telomere, concussion, prefrontal cortex, hippocampus, therapeutic

## Abstract

Children and adolescents have the highest rates of traumatic brain injury (TBI), with mild TBI (mTBI) accounting for most of these injuries. This demographic also often suffers from post-injury symptomologies that may persist for months. Telomere length (TL) has previously been used as a marker for outcomes following repetitive mild TBI (RmTBI) and it may be possible that telomere elongation can reduce post-traumatic behavioral impairments. Telomerase activator-65 (TA-65) is a telomerase small-molecule activator purified from the root of Chinese herbs that has been anecdotally reported to have anti-aging and life-extending potential. We hypothesized that RmTBI would shorten TL but administration of TA-65 would reverse RmTBI-induced telomere shortening and behavioral deficits. Male and female Sprague-Dawley rats were orally administered TA-65 or a placebo substance for 30 consecutive days [postnatal day (P) 25–55]. Following the injury protocol (mTBIs on P33, 36, and 40), rats went through a behavioral test battery designed to examine symptomologies commonly associated with mTBI (balance and motor coordination, exploratory behavior, short-term working memory, and anxiety- and depressive-like behaviors). TL in ear and brain tissue (prefrontal cortex and hippocampus) and relative expression of *TERT* and *Tep1* via qPCR were assessed 15 days following the last injury. We observed a heterogenous response between males and females, with TA65 administration resulting in increased mRNA expression of *TERT* and *Tep1* in female rats that experienced RmTBI, which was accompanied by some functional recovery on motor behavior and footslips in the beam walk task and depressive-like behavior in the forced swim task.

## Introduction

Traumatic brain injury (TBI) is a major public health issue and is one of the most common causes of death and disability in childhood and adolescence (1). Mild TBI (mTBI), or concussion, has been recently spotlighted within the media and accounts for 80% of all TBI's (2). The adolescent age group is at particularity high risk for mTBI, with male adolescents experiencing more mTBIs than females (3). Of all adolescent mTBIs, sport accounts for >60% of reported injuries (4). Alarmingly, adolescents are at particularly high risk for chronic post-injury deficits (5) and the long-term consequences of repetitive mTBI (RmTBI) during this critical period of brain development are largely unknown. However, recent adult literature has linked RmTBI to prolonged neurocognitive and behavioral changes, worse prognoses and long-term neurological sequelae, and poorer executive function, depression scores, and cognitive changes that have been related to the number of injuries received (6, 7).

The use of telomere length (TL) as a marker for outcomes following RmTBI has recently been explored within the literature (8). Hehar and colleagues found that shorter TL was associated with history of an mTBI and was also associated with worse performance on a behavioral test battery measuring, cognition, memory, anxiety-like, and depressive-like symptomologies (9). Wright and colleagues also found characteristic TL shortening associated with RmTBI, and these RmTBI-induced changes in TL were correlated with diffusion weighted MRI changes (8). These two studies suggest that TL may be a suitable biomarker for mTBI outcomes in rodent models.

Telomeres are evolutionary conserved DNA sequences (consisting of 6 bp repeats, TTAGGG) that act as capping structures for linear chromosomes (10). Telomeres have four main roles: distinguishing and protecting chromosomal ends, serving as a docking site for DNA repair proteins, and they provide the cell with important information regarding its proliferation history (10). While cellular division is the primary mechanism of telomere shortening, oxidative stress and inflammation are also significant sources of telomere loss (10–12). It is generally accepted that following each cell division, telomeres are shortened by ~50–150 bp (13). In both humans and rodents, telomere attrition is a well-associated marker of aging, although there are high degrees of interindividual differences (14). A number of genetic and environmental factors have also been shown to alter TL such as, exercise, diet, stress, and inflammation (15, 16). More recently, evidence has also demonstrated that a number of chronic diseases, as well as, a history of RmTBI, can significantly reduce TL (8, 9). Although TL is often discussed in the context of shortening, increases in TL are also biologically consequential, with many cancer cells exhibiting elongation of telomeres, which results in cellular immortalization (17). Therefore, optimal TL is delicate balance between processes that promote shortening (i.e., end-replication) and processes that promote lengthening (i.e., telomerase) (18).

Telomerase is a ribonucleoprotein complex responsible for extending telomeres by adding 6 base-pair repeats to the ends of chromosomes (19). The telomerase complex is a large tightly regulated molecule (~1 kDa) with many associated proteins (20). Two of the most important genes that code for the telomerase complex are telomerase reverse transcriptase (*TERT*) and telomerase-associated protein-1 (*Tep1*) (19). *TERT* codes for the catalytic subunit of telomerase which acts as the rate-limiting enzyme in telomerase activity (21). Without *TERT*, telomeres would shorten, and cells undergo cellular senescence or apoptosis (22). The addition of *TERT* to normally functioning cells increases activity of telomerase and therefore, increase TL (23), while humans with mutations in *TERT* gene have shorter telomeres and reduced telomerase activity (24). The second of these genes, *Tep1*, is associated with both telomerase RNA and *TERT*. *Tep1* is important for catalyzing the addition of new telomeres (21). One of the main functions of *Tep1* is the binding of TERT and the potential modulation of enzymatic activity (25, 26).

Telomerase activator-65 (TA-65) (also known as cycloastragenol), is a potent telomerase small-molecule activator purified from the root of Chinese herbs that has demonstrated ability to lengthen telomeres (27). Although un-validated, TA65 has been anecdotally and controversially touted to have anti-aging, and life-extending potential. TA65 can be orally administered as it undergoes extensive first-pass hepatic metabolism after being efficiently absorbed through the intestinal epithelium (27). In mice, TA65 has been able to rescue short telomeres in adult, older females, and haploinsufficent mouse embryonic fibroblasts (14). Additionally, in human studies, low doses of TA-65 was able to increase telomere length in older cytomegalovirus (CMV^+^) patients (28). Moreover, multiple pre-clinical studies have demonstrated that reactivation of telomerase in telomerase deficient mice improved cognitive function, modulated molecular outcomes, and even reduced neurodegeneration (14, 29, 30).

Given that prior research has demonstrated that shorter telomeres are associated with a history of mTBI and poorer behavioral outcomes, and that activation of telomerase improves cognition, it may be possible that telomere elongation can reduce behavioral impairments and some of the adverse sequelae associated with RmTBI. Moreover, as previous research has also demonstrated sex differences in RmTBI-induced TL shortening and *TERT* mRNA expression changes (31–33), we hypothesize that behavioral and molecular outcomes will be dependent upon sex. Therefore, the purpose of this study was to determine if TA-65 administration could recover the behavioral and genetic deficits associated with RmTBI. We administered TA-65 or a placebo substance to male and female adolescent rats prior to, and post RmTBI. We assessed telomere length in ear tissue and brain tissue [prefrontal cortex (PFC) and hippocampus (HPC)] following treatment and injuries, as well as relative expression of *TERT* and *Tep1* via qPCR. We hypothesized that RmTBI would shorten TL, but administration of TA-65 would reverse RmTBI-induced telomere shortening and behavioral deficits. Although preliminary, we demonstrate that the TA65-induced activation of telomerase may be a valuable strategy to promote recovery following RmTBI offering some benefit to females; decreasing hind leg footslips and depressive-like behavior in the forced swim task, while increasing TL and mRNA expression of telomerase related genes.

## Methods

Thirty-four male and female Sprague-Dawley rats were randomly assigned to one of four conditions, RmTBI + TA65 (*n* = 10), RmTBI + Placebo (*n* = 8), Sham + TA65 (*n* = 10), and Sham + Placebo (*n* = 6). All rats were bred in-house to 6 dams, weaned at postnatal day 21 (P21), and housed in groups of three or four. All rats were housed in an animal husbandry room at 21°C with a 12:12 light:dark cycle (lights on at 07:00, off at 19:00). The animals had *ad libitum* access to food and water.

### TA65 Administration

TA65 (TA Sciences Inc, Lexington, USA) was administered daily from P25-55 in Kraft peanut butter at a dose of 25 mg/kg. Placebo animals received the same daily amount of Kraft peanut butter, but without drug. This dosage was selected as previous *in vivo* literature had demonstrated potent telomerase activation when orally administered (14).

### RmTBI

Rats in both the TA65 and placebo groups were randomly allocated to receive either three mTBIs with the lateral impact (LI) device, as described in Mychasiuk et al. (31), or three sham injuries at P33, P36, and P40. Animals were anesthetized with isoflurane until a toe pinch drew no response. Animals were then placed in a prone position on a Teflon® board. A small weight (50 g) was pneumatically fired with an average speed of 9.03 m/s, or ~83.10 G, at the rat's head. The weight impacted a small magnetic aluminum plate that acted as a helmet. The aluminum plate protects the animal from skull damage, while the force of the weight impacting the plate propels the rat into a 180° horizontal rotation. Immediately following the injury, rats were treated with lidocaine and placed on heating pads in a supine position. Rats in the sham condition were anesthetized and treated with lidocaine, but did not receive an injury. The time-to-right, time to move from a supine to prone position, was used as a measure of loss of consciousness (31).

### Behavioral Testing

Following RmTBI or sham injury all rats underwent a behavioral test battery to assess post-concussive symptomology. This behavioral paradigm has been employed extensively in our laboratory as it is ethologically representative of the typical trajectory of post-concussive symptomology experienced by adolescent populations (6, 31, 33–35).

#### Beam Walking

Twenty-four hours following the 1st and 3rd injury, rats underwent the beam walk test to measure motor coordination (36). Rats were placed on the end of a tapered 165 cm beam suspended 1 m above the ground, with their home cage placed on the far end of the beam. The beam was fitted with a 2 cm ledge to catch the rat's legs from slips and prevent falling. The rats underwent five trials (the first unscored as a training trial). Rats were scored for their average time to cross the beam and total hind-leg foot slips that touched the safety ledge.

#### Open Field

Two-days following the 3rd injury, post-injury day 2, (PID2) rats were tested on measures of locomotor and exploratory behaviors in the open field in a well-lit room (Lux = 580) (37). Rats were placed in the middle of a 143 cm circular arena for 10 min. An overhead Ethos Vision camera tracked total distance traveled and time spent in the middle of the arena using Noldus EthoVision XT10 software.

#### Elevated Plus Maze (EPM)

On PID3, animals were tested for anxiety-like behavior with the EPM in a well-lit room. The EPM was constructed of black Plexiglas, elevated 55 cm above the ground, and contained two crossed open arms and two closed arms (Lux open Arms = 690, Lux closed arms = 360). The rats were placed in the center of the maze and filmed for 5 min. A research associate blinded to the experimental paradigm scored the time spent in the closed and open arms.

#### Novel Context Mismatch (NCM)

The NCM was conducted on P1D6-9. We utilized a modified version of the NCM as noted in Spanswick and Sutherland (38). Rats underwent three training sessions on PI6, PI7, and PI8. On the training days rats were placed in two contexts for 5 min. Context A, a clear plastic rectangular bin (70 × 40 × 33 cm) containing two identical objects (beer bottles). Context B, an opaque circular bin (36 cm high and a diameter of 47 cm) containing a different pair of objects (candle holders). The probe day occurred on PI9. During this day, rats were placed in Context A for 5 min, Context B for 5 min, home-cage for 5 min, followed by the Novel context for 5 min. The novel context environment was a modified Context B with one object from Context A and one object from Context B. The exploration time was recorded by measuring the time spent with each object. Investigation proportion was measured by taking the total time spent exploring the novel object, divided by the time exploring the novel and familiar object.

#### Forced Swim

The forced swim paradigm was implemented on PID14 as a measure of depressive-like behavior (39). We utilize a modified version of the forced swim task [similar to (40)]. Rats were placed in a 30 × 60 cm circular tube filled with room temperature water (~25°C) for 7 min. The water level was high enough so the rat's tail was not able to reach the bottom of the tank. After completion of the test, rats were dried and returned to their home cages. The water was changed in the tank between cages. All trials were videotaped and the 7 min session was scored for the time spent immobile by a research associate blind to experimental conditions.

### mRNA Analysis

Rats were euthanized at PID15 (P55) upon completion of all behavioral testing. Rats were anesthetized with isoflurane and quickly decapitated. Using the Zilles atlas (41), tissue from the PFC and HPC was extracted, immediately flash frozen on dry ice, and stored at −80°C until analysis. RNA and DNA were extracted from brain tissue according to manufacturer protocols using Allprep RNA/DNA Mini Kit (Qiagen, Germany). The purity and concentration were tested with a NanoDrop™ 2000 (ThermoFisher Scientific, USA). Purified RNA (2 μg) was reverse transcribed into cDNA using oligo(dT) 20 Superscript III First-Strand Synthesis Supermix Kit (Invitrogen, USA).

Two genes were chosen for analysis for their importance in telomerase functioning.

Telomerase (*TERT)* and telomerase associated protein (*Tep1*). For qRT-PCR, 10 ng of cDNA sample, 0.5 uM of the forward primer, 0.5 uM of the reverse primer, and 1X SYBR Green FastMix was loaded into each plate well. Primers for the qRT-PCR were designed using Primer3 (http://bioinfo.ut.ee/primer3) and purchased from Integrated DNA Technologies (Coralville, USA). Duplicate samples were run in 96-well plates for each gene. qRT-PCR was run with CFX Connect Real-Time PCR detection system (Bio-Rad, Hercules, CA, USA). Relative gene expression was normalized against two housekeeping genes, *Ywhaz* and *CycA* using the 2^ΔΔCt^ [as described by Pfaffl (42)].

### Telomere Length Analysis

Ear notches were taken at two time points, P33 (prior to mTBI #1) and P55 (euthanasia). Tissue was also taken from the PFC and HPC at P55. All tissue was stored at −80°C until analysis. Genomic DNA was extracted from tissue using Sigma RedExtract N-Amp Tissue PCR Kit according to manufacturer's specifications. The quantity and quality of DNA was measured with NanoDrop™ 2000 (ThermoFisher Scientific, USA). To conduct analysis genomic DNA was diluted to a concentration of 10 ng/ul. Each reaction required 1 ul of diluted genomic DNA in 20 ul 1X SYBR Green FastMix with Rox for qRT-PCR. Primers for 36B4 and Tel were designed using Primer3 and ordered from Integrated DNA technologies.

Primers were used at a concentration of 20 uM for the forward and reverse primer for both 36B4 and Tel. Two no-template controls were run on each plate. Each sample was run in duplicate on a 96 well-plate using the CFX Connect Real-Time PCR detection system (Bio-Rad, Hercules, CA, USA). Telomere length was determined by comparing the telomere to single copy ratio (Tel/36B4). The Tel/36B4 ratio was determined with a linear regression equation from (43), *y* = 1,910.5 × + 4,157, where, *y* = telomere length and *x* = −2^ΔCt^. The change in telomere length was determined by comparing the telomere length at sacrifice from TBI.

### Statistical Analysis

All statistical analyses were carried out using SPSS 25.0 for MAC. Three-way ANOVAs with Sex (Male: Female), Injury (RmTBI: Sham), and Treatment (TA65: Placebo), as factors were run for all behavioral and molecular results. *Post-hoc* pairwise comparisons (LSD) were performed where applicable to further examine significant interaction effects. *p'*s < 0.05 were considered statistically significant, and all graphs display means ± standard error. All data will be made available upon request to the corresponding author.

## Results

### Animal Characteristics

The three-way ANOVA for weight gained between the first mTBI (P30) and the end of the experiment (P55) demonstrated that there were no significant differences associated with injury (*p* = 0.85) or treatment (*p* = 0.97). There was however a significant difference in weight gained between males and females (*p* < 0.01). The three-way ANOVA for brain weight at euthanasia, also found no significant main effects of treatment or injury (*p*'s > 0.05), but also revealed a significant sex effect, whereby male brains were heavier than female brains (*p* < 0.01).

### Behavioral Measures

**Figure 1:**
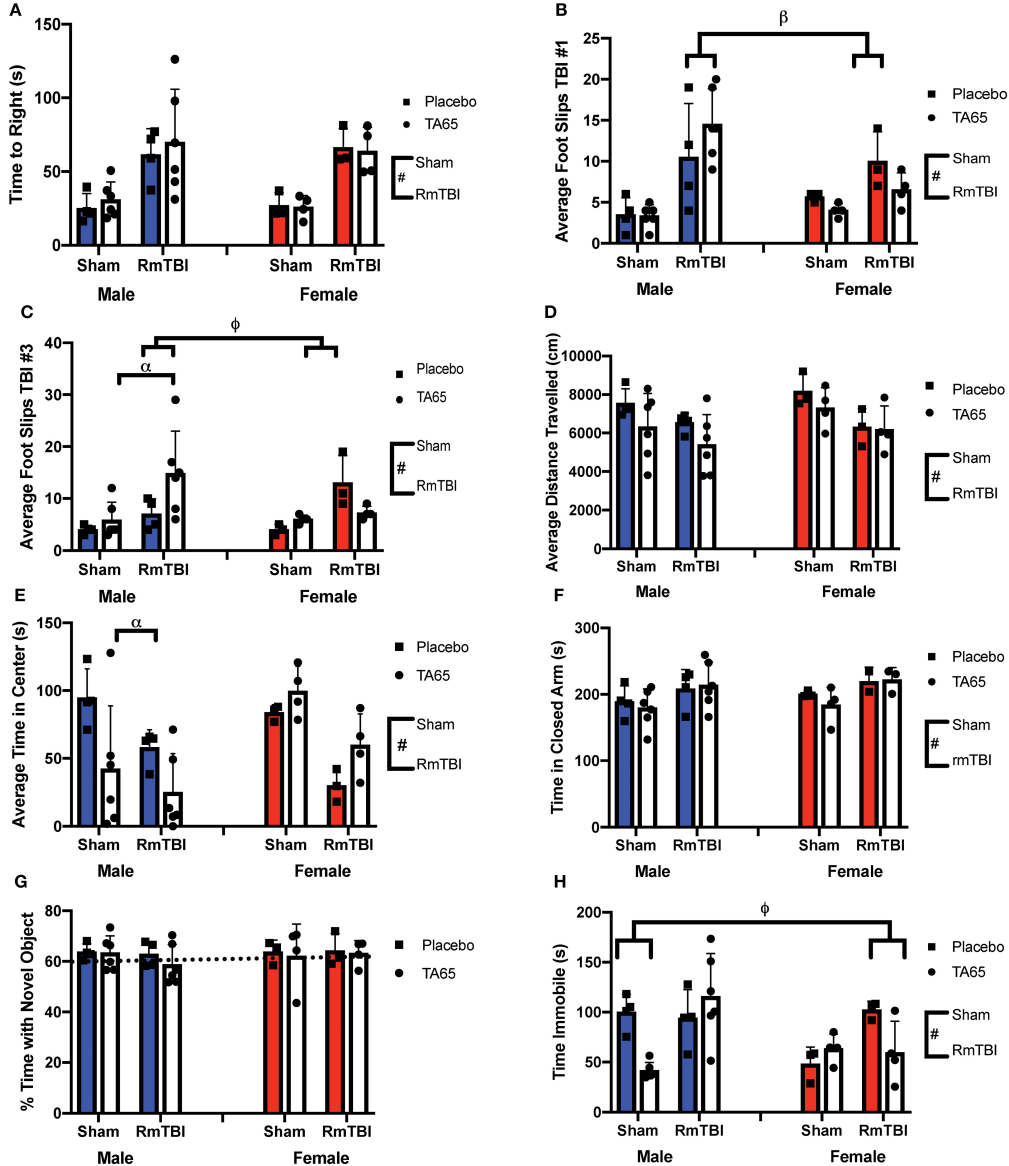
Graphical representation of behavioral testing displayed with means ± SEM. Solid colored bars indicate placebo treatment, while white bars indicate TA65 treatment. (#) Indicates a main effect for RmTBI, (*) indicates a main effect for sex, (α) indicates a sex*treatment interaction, (β) indicates a sex*RmTBI interaction, (ϕ) indicates a sex*treatment*RmTBI interaction. **(A)** Time to right. **(B)** Average number of foot slips following TBI #1 whereby males who received RmTBI had more foots slips than females who received RmTBI. **(C)** Average number of foot slips following TBI #3 whereby males who received TA65 had more foot slips than placebo males. Furthermore, RmTBI males who received TA65 had more foot slips than RmTBI females who received TA65. **(D)** Average distance traveled in the open field. **(E)** Total time spent in the center of the open field, whereby males who received TA65 had less time spent in the center compared to placebo males. **(F)** Time spent in the closed arms of the EPM. **(G)** Percent time spent investigating the novel object in NCM. The dashed line indicates chance performance on the task. **(H)** Time spent immobile in forced swim, whereby sham males treated with TA65 spent less time immobile than sham placebo males, and RmTBI females treated with placebo had more time immobile than RmTBI females treated with TA65.

Statistical results from the three-way ANOVAs for our behavioral test battery are represented in Table 1 (graphically in Figure 1). To summarize, we identified one main effect of sex in the forced swim task (Figure 1H), whereby males exhibited an increased time immobile when compared to females. Consistent with previous studies in our laboratory, we identified a main effect of injury on 7/8 measures. RmTBI was associated with an increase in loss of consciousness, increased motor deficits, decreased locomotor ability, decreased exploratory behavior as measured with time spent in the center of open field, increased anxiety-like behavior, and increased depressive-like behavior. We failed to identify any main effects of treatment, although there were two trends toward significance in total distance traveled in the open field and time immobile in the forced swim task. However, there were numerous significant interactions, many of which involved the treatment condition, that are discussed below.

**Table 1:** Statistical analysis for the behavioral tests of three-way ANOVA's with main effects of sex, drug, RmTBI in adolescent rats.

**Behavior test**	**Effect of sex** ***F* (p)**	**Effect of drug** ***F* (p)**	**Effect of injury** ***F* (p)**	**Drug x sex** ***F* (p)**	**Sex x injury** ***F* (p)**	**Drug × injury** ***F* (p)**	**Drug × sex × injury** ***F* (p)**
Time-to-right	0.02 (0.88)	0.16 (0.70)	30.53 (<0.01)	0.41 (0.53)	0.01 (0.96)	0.01 (0.96)	0.02 (0.88)
Beam walk #1	1.46 (0.24)	0.08 (0.78)	28.36 (<0.01)	3.68 (0.07)	5.83 (<0.05)	0.25 (0.62)	1.63 (0.21)
Beam walk #2	0.05 (0.82)	0.94 (0.34)	13.29 (<0.01)	4.83 (<0.05)	0.08 (0.78)	0.08 (0.78)	5.07 (<0.05)
Open field distance	1.47 (0.24)	3.58 (0.07)	7.65 (<0.01)	0.59 (0.45)	0.36 (0.56)	0.21 (0.65)	0.15 (0.70)
Open field center time	1.87 (0.18)	1.04 (0.32)	14.26 (<0.01)	11.29 (<0.01)	1.04 (0.32)	0.73 (0.40)	0.02 (0.89)
EPM	0.74 (0.40)	0.20 (0.66)	7.31 (<0.05)	0.06 (0.81)	0.01 (0.94)	0.70 (0.41)	0.01 (0.91)
NCM	0.21 (0.65)	0.49 (0.49)	0.16 (0.70)	0.04 (0.85)	0.49 (0.49)	0.09 (0.77)	0.19 (0.67)
Forced swim	4.54 (<0.05)	3.09 (0.09)	10.42 (<0.01)	0.061 (0.81)	0.24 (0.63)	0.36 (0.55)	14.24 (<0.01)

#### *Post-hoc* Analysis of Behavioral Measures

In the first beam walk task, following the first mTBI, there was a significant sex by injury interaction, where *post-hoc* analysis revealed that mTBI males exhibited more foot slips than females (*p* < 0.01) (Figure 1B). In the second beam walk task, 1 day following the 3rd injury, there was a significant three-way interaction. In this instance, *post-hoc* analysis demonstrated that while TA65 treatment reduced footslips in females that had experienced RmTBI, TA65 treatment actually exacerbated motor deficits in males who received RmTBI (*p* < 0.01) (Figure 1C). With respect to time spent in the center of the open field, there was a significant sex by treatment interaction, whereby males who received TA65 treatment actually displayed increased anxiety as measured by less time spent in the center of the arena (*p* < 0.01) (Figure 1E). Finally, a three-way interaction was also identified in the forced swim paradigm. *Post-hoc* analysis demonstrated that for females with RmTBI, TA65 treatment reduced TBI-induced depressive-like behavior (*p* < 0.05), whereas for males, TA65 treatment reduced depressive-like behaviors sham animals (*p* < 0.01) (Figure 1H). In summary, it appears that TA65 treatment exacerbated behavioral symptomologies in male rodents (footslips and time in the center of the open field), while offering some benefit to females with RmTBI (footslips and depressive-like behavior in the forced swim task).

### Molecular Measures

**Figure 2:**
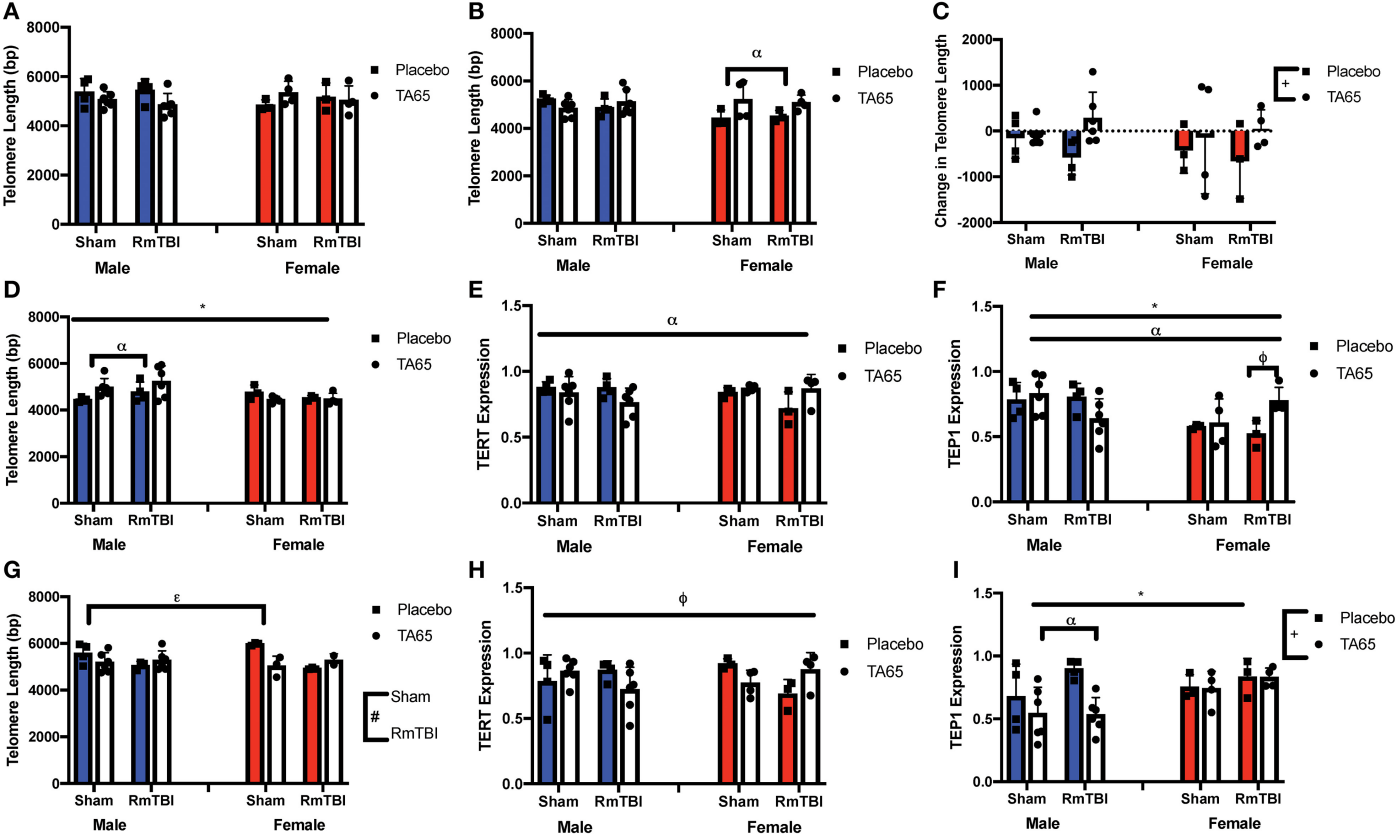
Graphical representation of molecular measures displayed as mean ± SEM. (#) Indicates a main effect for RmTBI, (*) indicates a main effect for sex. (+) indicates a main effect for treatment, (α) indicates a sex*treatment interaction, (ε) indicates a treatment*RmTBI interaction, (ϕ) indicates a three-way interaction. **(A)** Average Ear Notch TL prior to treatment and injuries. **(B)** Average Ear Notch TL at sacrifice, whereby females treated with TA65 had longer telomeres. **(C)** Change in Ear Notch TL, whereby treatment with TA65 elongated telomeres. **(D)** TL in the PFC showed males had longer TL and males treated with TA65 had longer TL than males treated with placebo. **(E)** Relative expression of *TERT* in the PFC indicates a sex*treatment interaction, although *post-hoc* analysis did not reveal a significant difference. **(F)** Relative expression of *Tep1* in the PFC, whereby *post-hoc* analyses demonstrated that females treated with TA65 and sustaining an RmTBI experienced higher *Tep1* expression (*p* < 0.05). **(G)** TL in the HPC, with *post-hoc* analysis demonstrating that sham animals treated with TA65 having longer TL than sham animals treated with placebo (*p* < 0.05). **(H)** Relative *TERT* expression in the HPC with *post-hoc* analysis indicating that placebo females receiving RmTBI experienced lower expression of *TERT* (*p* < 0.05). **(I)** Relative expression of *Tep1* in the HPC with males treated with TA65 having lower expression of *Tep1* (*p* < 0.01).

Statistical results from the three-way ANOVAs for the molecular analysis is represented in Table 2 (graphically in Figure 2). As expected, there were no group differences in telomere length at the original sample collection time indicating that the groups were not different prior to treatment (Figure 2A). Although we failed to replicate prior studies demonstrating that RmTBI reduces ear notch TL and PFC TL (Figures 2B,D, respectively), we did demonstrate that RmTBI resulted in reductions in TL in the HPC (Figure 2G). However, the inability to identify significant losses of TL may have been because TA65 treatment increased ear notch TL in females (Figure 1B), and PFC TL in males (*p* < 0.05) (Figure 1D). In further support of this, when examining change in ear notch TL (telomere length at sacrifice—telomere length at baseline), there was a main effect of treatment indicating that TA65 did in fact attenuate normal reductions in telomere length over time (*p* < 0.05) (Figure 2C).

**Table 2:** Statistical results for the three-way ANOVAs for telomere length obtained from ear notches, PFC, and HPC, as well as mRNA expression in PFC and HPC.

**Molecular test**	**Effect of sex** ***F* (p)**	**Effect of drug** ***F* (p)**	**Effect of injury** ***F* (p)**	**Drug × sex** ***F* (p)**	**Sex × injury** ***F* (p)**	**Drug × injury** ***F* (p)**	**Drug × sex × injury** ***F* (p)**
Original Telomere	0.27 (0.61)	0.61 (0.44)	0.04 (0.85)	3.62 (0.07)	0.07 (0.80)	1.92 (0.18)	0.25 (0.62)
Sacrifice Telomere	1.67 (0.21)	3.51 (0.07)	0.03 (0.86)	5.47 (<0.05)	0.01 (0.95)	0.42 (0.52)	1.73 (0.20)
Delta Telomere	0.54 (0.47)	4.54 (<0.05)	0.03 (0.86)	0.01 (0.94)	0.01 (0.97)	1.75 (0.20)	0.15 (0.70)
PFC Telomere	4.79 (<0.05)	1.24 (0.28)	0.39 (0.54)	5.79 (<0.05)	2.12 (0.16)	0.12 (0.74)	0.38 (0.54)
HPC Telomere	0.06 (0.82)	1.92 (0.18)	4.89 (<0.05)	0.58 (0.45)	0.41 (0.53)	11.78 (<0.01)	1.56 (0.27)
*TERT* PFC	0.19 (0.67)	0.03 (0.86)	2.25 (0.15)	6.28 (<0.05)	0.16 (0.70)	0.12 (0.73)	1.93 (0.18)
*TEP1* PFC	8.88 (<0.01)	0.71 (0.41)	0.09 (0.77)	4.31 (<0.05)	2.24 (0.15)	0.01 (0.94)	5.28 (<0.05)
*TERT* HPC	0.01 (0.94)	0.03 (0.87)	1.01 (0.32)	0.33 (0.57)	0.16 (0.69)	0.34 (0.57)	9.32 (<0.01)
*TEP1* HPC	4.93 (<0.05)	5.09 (<0.05)	2.76 (0.11)	4.55 (<0.05)	0.03 (0.86)	0.96 (0.37)	1.10 (0.30)

As TA65 is believed to activate telomerase, we also examined PFC and HPC expression of two genes that encode critical proteins for telomerase, *TERT* and *Tep1*. In both the PFC and HPC, *post-hoc* analysis of the significant treatment by sex interaction indicated that treatment with TA65 reduced *TERT* mRNA expression in males who experienced RmTBI, but increased *TERT* expression in females who experienced RmTBI (Figures 2E,H). For *Tep1* expression significant sex effects were identified in both the PFC and HPC, however the results were opposite (*p*'s < 0.05). In the PFC males exhibited higher *Tep1* mRNA expression than females (Figure 2F), however in the HPC, females exhibited higher levels than males (Figure 2I). In the PFC there was also a significant three-way interaction for *Tep1* mRNA expression, with *post-hoc* analysis demonstrating that TA65 treatment reduced *Tep1* levels in males that had experienced RmTBI, while increasing expression of *Tep1* in females with RmTBI (*p* < 0.05) (Figure 2F). Finally, in the HPC, *post-hoc* analysis of the significant treatment by sex interaction demonstrated that males who received TA65 had lower *Tep1* mRNA expression when compared to males treated with the placebo (*p* < 0.01) (Figure 2I). Similar to the results from the behavioral measures, it appears that TA65 treatment provided greater benefit to female animals as compared to males.

## Discussion

The goal of the current study was to determine if administration of TA65 could increase telomerase activity and TL, thereby improving functional recovery from RmTBI. We found that TA65 treatment exacerbated some behavioral symptomologies in male rodents (impaired balance and motor coordination, increased anxiety-like behavior), while offering a small benefit to females with RmTBI (improved motor coordination and reduced depressive-like behavior). With respect to TL, we found that TA65 treatment increased ear notch TL in females, and PFC TL in males, while also attenuating normal reductions in telomere length over time. Finally, in females with RmTBI, but not males, TA65 increased expression of the genes that code for the telomerase complex. In summary, TA65 administration resulted in increased mRNA expression of *TERT* and *Tep1* in female rats that experienced RmTBI, and these rats also exhibited some functional benefit, as measured with our behavioral paradigm.

Even within the healthy brain both glia and neurons are susceptible to significant telomere shortening. Glial cells because they are mitotic, and neurons (although post-mitotic) are excitable and therefore exhibit higher metabolic rates and increased iron/copper content, which subsequently leads to higher levels of oxidative stress (44, 45). Under normal conditions, cells have mechanisms dedicated to adequately manage oxidative stress and reactive oxidative species (ROS). However, following TBI, cells may be overwhelmed and unable to compensate for the added cellular damage. The “secondary injury,” which occurs after the initial biomechanical injury, is a delayed and protracted period of damaging processes that included excitotoxicity, oxidative stress, apoptosis, and mitochondrial dysfunction (46). These secondary injury processes often lead to an accumulation of ROS which have been associated with significant DNA damage (47, 48). Consistent with this, in the HPC of this study and in numerous previous studies, we have demonstrated that mTBI and RmTBI reduce TL (8, 9), likely associated with increased oxidative stress and DNA damage. As DNA was extracted from all HPC and PFC tissue, we are unable to conclude if the changes in TL are associated with neuronal or glial populations, but given that activation of microglia significantly represses expression of telomerase associated genes (49), cell sorting would be an important next step toward understanding the mechanisms of telomere shortening in response to TBI.

While activation of telomerase and manipulation of TL is associated with obvious benefits, such as reducing DNA damage and increasing the probability of adequate DNA repair, changes in TL are also associated with changes in gene expression. The reversible silencing of genes near telomeres, characterized as the Telomere Position Effect (TPE), involves conformational changes in chromatin that leads to silencing of genes based on the length of the telomere and their distance from the telomere (50), and may be responsible for silencing or activating genes important for neuroplasticity and repair. For example, genes such as sonic hedgehog (*SHH*) (51) which is involved in neuroplasticity, neurotrophin 3 (*NT-3*) which plays a role in neuronal survival (52), kruppel like factor 6 (*KLF6*) involved in axonal regrowth following injury (53), and glutathione peroxidase 4 (*GPX4*) an antioxidant gene (54), are located at the ends of chromosomes and would be susceptible to TL-dependent silencing. While beyond the scope of this study, future investigation could examine if certain chromosomes are more susceptible to TBI-induced telomere attrition, as this could provide valuable insight into TPE silencing of genes critical to recovery and repair.

Finally, although we often identify sex differences in the context of RmTBI (55–58), the striking divergence in TA65 treatment efficacy for males and females within this study is surprising. Not only did TA65 fail to offer any benefit to males, it actually exacerbated many of the outcomes. This could have been a consequence of the interaction between sex hormones and oxidative stress. Estrogen is known to be a potent antioxidant, to regulate the expression of many antioxidant genes, and reduce the production of ROS (59), while testosterone increases susceptibility to oxidative stress and has no known antioxidant properties (60). In addition, estrogen has been shown to directly activate the telomerase promotor (61). It may be possible that TA65 acted on this native telomerase activation mechanism, compounding the telomere lengthening effects of estrogen in females. The effect of TA-65 on males, who do not normally exhibit the estrogen mediated mechanism, may have been too minor or may not have exceeded a threshold for promotor input required to achieve noteworthy telomerase activation. Future studies should investigate whether or not a higher dose of TA65 are able to produce benefit in males as well, and whether or not, these sex differences are in fact driven by estrogen.

In conclusion, although preliminary, this study provides evidence that activation of telomerase may be a valuable strategy to promote recovery following RmTBI. This is consistent with growing evidence that therapeutically targeting DNA damage is a viable mechanism to improve neurological deficits (45). However, there are numerous limitations within this study that require further investigation. First, the profound sex differences warrant examination; is TA65 detrimental for males under all conditions or is there an optimal dosage or timing paradigm that would prove efficacious. Second, this study provided TA65 throughout the experiment, and it would be advantageous to examine its effectiveness when administered only prior to, or following the traumatic events. And finally, animals within this study were euthanized at a young age (P55). Future studies should examine long-term outcomes to ensure that administration of a telomerase activator does not increase susceptibility to / or risk for cancer and cancer-related disorders.

## Data Availability Statement

The datasets generated for this study are available on request to the corresponding author.

## Ethics Statement

The animal study was reviewed and approved by Canadian Council of Animal Care and received approval from the University of Calgary Conjoint Facilities Ethics Approval Board.

## Author Contributions

EE was involved in data collection, analysis, and writing of the manuscript. HM was involved in data collection and writing of the manuscript. RM was responsible for experimental design, data collection, analysis, and writing of the manuscript.

### Conflict of Interest

The authors declare that the research was conducted in the absence of any commercial or financial relationships that could be construed as a potential conflict of interest.
